# Acute Extrapyramidal Side Effects Associated With the Combined Use of Low Doses of Haloperidol and Clarithromycin

**DOI:** 10.7759/cureus.51020

**Published:** 2023-12-24

**Authors:** Serkan Gunes, Gupse Inal Ulutas, Pinar Sefik, Hamide Kubra Ozluk, Esra Erol Tanrikulu

**Affiliations:** 1 Child and Adolescent Psychiatry, Adana City Training Hospital, Adana, TUR

**Keywords:** eps, haloperidol, clarithromycin, side‐effect, typical antipsychotic, tic disorder and psychiatry

## Abstract

Extrapyramidal side effects (EPS) are one of the major side effects that may frequently occur in the use of antipsychotics. EPS may cause distress and worsen the psychopathological condition. In this paper, we report a case of a 12-year-old boy with tic disorders who developed EPS after using haloperidol and clarithromycin combined.

## Introduction

Antipsychotic medications are generally used in children and adolescents for bipolar disorder, depression, schizophrenia, anxiety disorders, and tic disorders. Like other drugs, antipsychotic agents may have both beneficial and adverse effects at the optimum dose used for treatment [1]. One of the most important adverse effects of antipsychotic medications is extrapyramidal side effects (EPS) such as dystonia, akathisia, parkinsonism, or tardive dyskinesia [2,3]. In this paper, we report the occurrence of acute EPS with haloperidol and clarithromycin use in a boy with tic disorders.

## Case presentation

**Figure 1:**
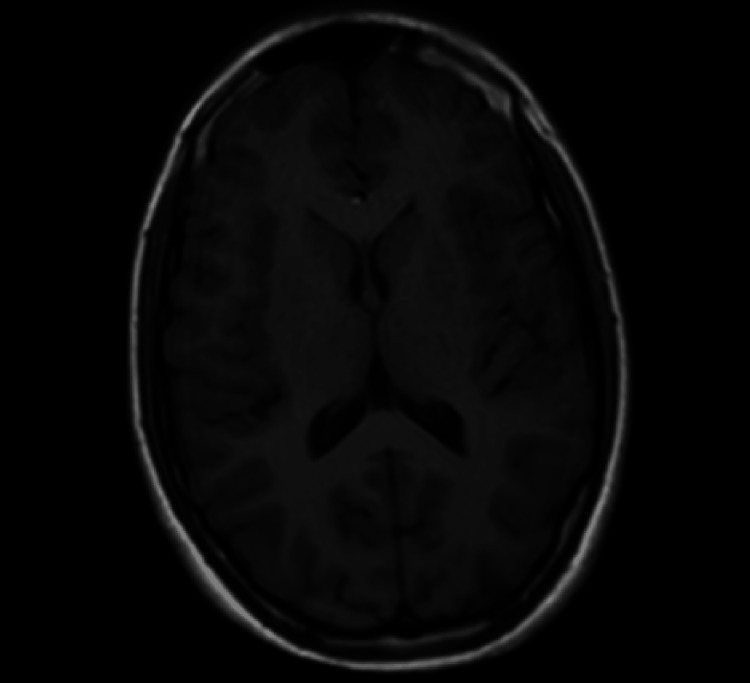
Normal brain MRI MRI: Magnetic resonance imaging

A 12-year-old boy was referred to our child and adolescent psychiatry clinic with involuntary motor movements. He was diagnosed with tic disorders two years ago and used haloperidol 2 mg/day for six months without any adverse effects. After two years, his involuntary motor movements started again and distressed the patient. During the clinical evaluation, we observed motor movements on his face, eyelids, and arms that were compatible with tic disorders. There was no physical illness or trauma in history. Brain magnetic resonance imaging (Figure 1), neurological examinations (mental status, motor and sensory skills, balance and coordination, reflexes) and laboratory tests (white blood cell count (WBC): 5.9 (3.84-9.84 10^3^/µl), hemoglobin: 14.4 (11-14.5 g/dL), thrombocytes: 297 (175-332 10^3^/µl), glucose: 89 (60-100 mg/dL), thyroid stimulating hormone (TSH): 0.812 (0.34-5.6 mUI/L), free T3: 4.1 (2.6-4.37 ng/L), blood urea nitrogen (BUN): 19 (17-43 mg/dL), creatinine: 0.50 (0.24-0.73 mg/dL), albumin: 43.7 (35-55 g/L), ferritin: 78.5 (23.9-336 µg/L), iron: 96 (50-120 µg/dL), direct bilirubin: 0.09 (0-0.2 mg/dL), total bilirubin: 0.37 (0.3-1.2 mg/dL), alanine aminotransferase (ALT): 17 (5-50 U/L), aspartate aminotransferase (AST): 22 (5-50 U/L)) were normal and haloperidol 0.5 mg/day was initiated gradually.

**Figure 2:**
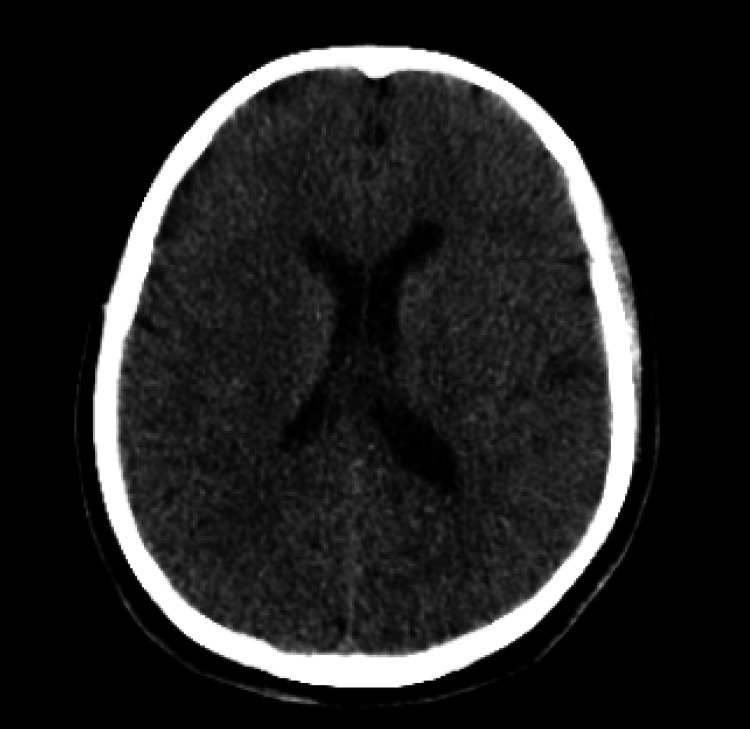
Normal brain CT CT: Computed tomography

The next day, he was admitted to our emergency service with dystonia, and difficulties in speech and swallowing. Brain computed tomography (Figure 2), neurological examinations, and laboratory tests (WBC: 9.1 (3.84-9.84 10^3^/µl), hemoglobin: 13.6 (11-14.5 g/dL), thrombocytes: 290 (175-332 10^3^/µl), glucose: 96 (60-100 mg/dL), TSH: 1.034 (0.34-5.6 mUI/L), free T3: 3.51 (2.6-4.37 ng/L), BUN: 17 (17-43 mg/dL), creatinine: 0.64 (0.24-0.73 mg/dL), ferritin: 72.4 (23.9-336 µg/L), iron: 90 (50-120 µg/dL), sodium: 141 (136-146 mmol/L), potassium: 4.25 (3.5-5.5 mmol/L), chloride: 103 (96-110 mmol/L), direct bilirubin: 0.13 (0-0.2 mg/dL), total bilirubin: 0.64 (0.3-1.2 mg/dL), ALT: 16 (5-50 U/L), AST: 21 (5-50 U/L), creatine kinase: 102 (5-171 U/L), C-reactive protein: 3 (0-8 mg/L)) in the emergency clinic were unremarkable. However, it was learned that the patient was also using 125 mg/day of clarithromycin for upper tract infection. The intramuscular injection of 5 mg/ml biperiden was applied to the patient and extrapyramidal symptoms were relieved in 30 minutes. The patient and his family members did not describe any side effects to their previous use of clarithromycin and he used 125 mg/day of clarithromycin for 10 days. After the patient's infection cleared, haloperidol 0.5 mg/day was restarted gradually and no side effects were observed despite the third month of treatment.

## Discussion

We reported a case who developed acute EPS after using low doses of haloperidol and clarithromycin combined and relieved after biperiden injection. The occurrence of EPS in the use of only these two drugs may indicate that EPS develops due to combined use. Also, the patient did not have any medical illnesses, trauma, or history of previous EPS.

When antipsychotics block dopamine (D2) receptors, dopamine cannot prevent acetylcholine release in the nigrostriatal region. This may lead to overactivity of acetylcholine in the basal ganglia which may cause EPS [4]. Dystonia, also seen in our case, is a type of EPS with sustained or intermittent involuntary muscle action. Especially in children and young adults, dystonia may occur in 25-40% of patients using first-generation antipsychotics (FGA) like haloperidol, chlorpromazine, or zuclopenthixol. Acute dystonia usually occurs between 24 and 48 hours of oral FGA use [5].

Haloperidol is a D2 receptor antagonist that can be used in child and adolescent psychiatry practice for a variety of clinical conditions, such as psychosis, mood disorders, or tic disorders [4]. It is metabolized in the liver primarily by cytochrome P450 3A enzyme (CYP3A4) and is subsequently cleared mainly by glucuronidation [6]. Clarithromycin is a macrolide antibacterial agent widely used in the treatment of respiratory tract infections and inhibits CYP3A in the liver [7]. In this context, a drug interaction between haloperidol and clarithromycin via CYP3A may cause increased serum levels of haloperidol and overall antipsychotic effects. Increased antipsychotic activity with this drug-drug interaction may be related to the emergence of EPS in our case.

The use of anticholinergic medications may reduce acetylcholine overactivity resulting from D2 receptor blockade. Therefore, administration of an anticholinergic like diphenhydramine, biperiden, or benztropine may reduce EPS caused by antipsychotics [4]. In emergencies, as shown in our case, studies have suggested that intramuscular anticholinergic injection may resolve EPS within 30 minutes [8].

## Conclusions

The combined use of antipsychotics and antibiotics has become widely common in clinical practice. Therefore, clinicians should be careful in their drug choices in order to reduce the possible adverse effects of drug interactions.
